# Analysis of potential categories of depression in older adults with chronic illness

**DOI:** 10.3389/fpsyg.2026.1701471

**Published:** 2026-01-27

**Authors:** Yiping Qu, Xiaomin Sun, Ya Zhao

**Affiliations:** The Third Hospital of Xi’an, Xi’an, Shaanxi, China

**Keywords:** CHARLS (China Health and Retirement Longitudinal Study), chronic illness, depression, older adults, potential categories

## Abstract

**Objective:**

This study aims to explore the prevalence of depression in older adults with chronic diseases and to identify heterogeneous subgroups within this population. Additionally, we seek to investigate the influencing factors associated with depressive conditions among older adults. This study aims to provide a foundational reference for the development of targeted intervention programs.

**Methods:**

This study utilized data from the 2018 China Health and Aging Tracking Survey (CHARLS), including 5,878 older adults with chronic diseases. We explored potential categories of depression among these individuals through latent profile analysis. Additionally, one-way chi-square tests and one-way ANOVA were employed for single factor, while multi-factor correlation was identified via multinomial logistic regression analysis.

**Results:**

A total of 5,878 older adults with chronic diseases were included in this study. The participants were categorized into four groups: low level (1,904; 32.4%), low level with high despair (304; 5.2%), medium level (2,963; 50.4%), and high level (707; 12.0%). Univariate analysis of variance revealed significant differences among all variables, except for the presence of only one child. In comparison to the low level group (i.e., control group), the factors influencing the high level group included anxiety, instrumental activities of daily living (IADL), living alone, self-assessment of current life and health, exercise, activity limitation, financial self-assessment, and marital status. For the medium level group significant factors included anxiety, living alone, health self-evaluation, alcohol use, exercise, and activity limitation. Influential factors for the low level with high despair group include health self-evaluation, exercise, engagement in regular physical labor, and economic self-assessment.

**Limitations:**

The CES-D-10 scale may not fully capture the presence of depression in older adults with high levels of chronic illness.

**Conclusion:**

Depression in older adults with chronic illnesses, stemming from both physical and psychological factors, can significantly impact their overall health status. Therefore, it is essential to provide more targeted healthcare services tailored to the diverse needs of this population in future community health initiatives. Such approaches aim to improve depressive symptoms and enhance the quality of life for older adults.

## 1 Introduction

Chronic diseases pose a significant burden on global health. Currently, chronic diseases account for over 36 million deaths worldwide, representing three-fifths of all fatalities, making them a critical public health issue that affects the global economy (World Health Organization, 2014). In China, which has the largest elderly population over the age of 60, approximately 180 million (75%) individuals suffer from chronic diseases (Huang et al., 2019). According to China’s medium- and long-term plan for the Prevention of Chronic Diseases (2017–2025), these conditions primarily include cardiovascular and cerebrovascular diseases, diabetes, chronic respiratory diseases such as chronic obstructive pulmonary disease, and others.

The prevalence of chronic diseases among the elderly not only significantly impacts their quality of life but also leads to psychological disturbances. Older adults with chronic illnesses may experience adverse psychological states, including depression and anxiety, particularly in high-stress situations. Compared to the general population, the elderly face a higher prevalence of chronic diseases and are more psychologically vulnerable. Research indicates that older adults with chronic conditions may experience anxiety and depression due to economic factors, psychological stress, and poor self-management capabilities, which can adversely affect daily life and hinder effective treatment and recovery (Hao et al., 2025).

Therefore, it is essential to assess the mental health status of older adults with chronic diseases, identify influencing factors, and provide targeted interventions to improve their psychological well-being, thereby promoting active aging. However, many previous studies have assessed psychological states based solely on total scores, typically reporting results as “yes” or “no” regarding depression or poor psychological health, which fails to account for the heterogeneity of psychological distress (Ghiggia et al., 2022). This one-dimensional assessment overlooks the individual differences among older adults with chronic diseases.

This study aims to explore the characteristics of psychological distress in older adults with chronic diseases using latent profile analysis (LPA), clarifying the relationship between latent categories of depressive states and their influencing factors. LPA is a person-centered approach, in contrast to variable-centered methods. Recent studies have utilized LPA to examine depressive states among older adults. For instance, Wang S. et al. (2023) conducted a latent profile analysis of cognitive functioning and depressive symptoms in Chinese older adults, identifying four subgroups related to cognitive impairment and depressive status: mild cognitive impairment, moderate cognitive impairment, mild cognitive impairment combined with depression, and moderate cognitive impairment combined with depression. This approach allows for a clearer understanding of the differences among subgroups within the study population.

Despite the insights from existing studies, there have been no reports applying LPA to investigate the depressive status of older adults with chronic diseases. Therefore, this study will utilize LPA to identify depressive subtypes in this population, clarify the relationship between each influencing factor and the potential categories of depression, and compare the differences in depressive statuses among these categories. Although existing research has confirmed the association between chronic diseases and depression, current studies still exhibit several limitations: First, most investigations treat depression as a single research issue, overlooking its heterogeneity and failing to identify subtypes with distinct clinical characteristics and prognoses (Ye et al., 2025). Second, large-scale, population-based studies targeting elderly individuals with chronic diseases in Chinese communities remain scarce. Furthermore, the application of advanced statistical methods such as latent class analysis in this field remains insufficient (Xin et al., 2025). The goal is to provide specific intervention recommendations in future studies to enhance the quality of life for older adults.

## 2 Measurement and methods

### 2.1 Sample

The data for this study was sourced from the China Health and Aging Tracking Survey (CHARLS), which includes survey results from 113,000 households collected between 1998 and 2018. This comprehensive survey has been conducted eight times across 23 regions, including provinces (autonomous regions), municipalities, and counties in China. Utilizing various methodologies, CHARLS provides a multifaceted assessment of the health status of older adults, making the data both representative and widely recognized by scholars both domestically and internationally. All research participants voluntarily signed informed consent forms. After removing private information, the data became available for free through the Peking University Open Research Data Platform.^1^

**Figure 1:**
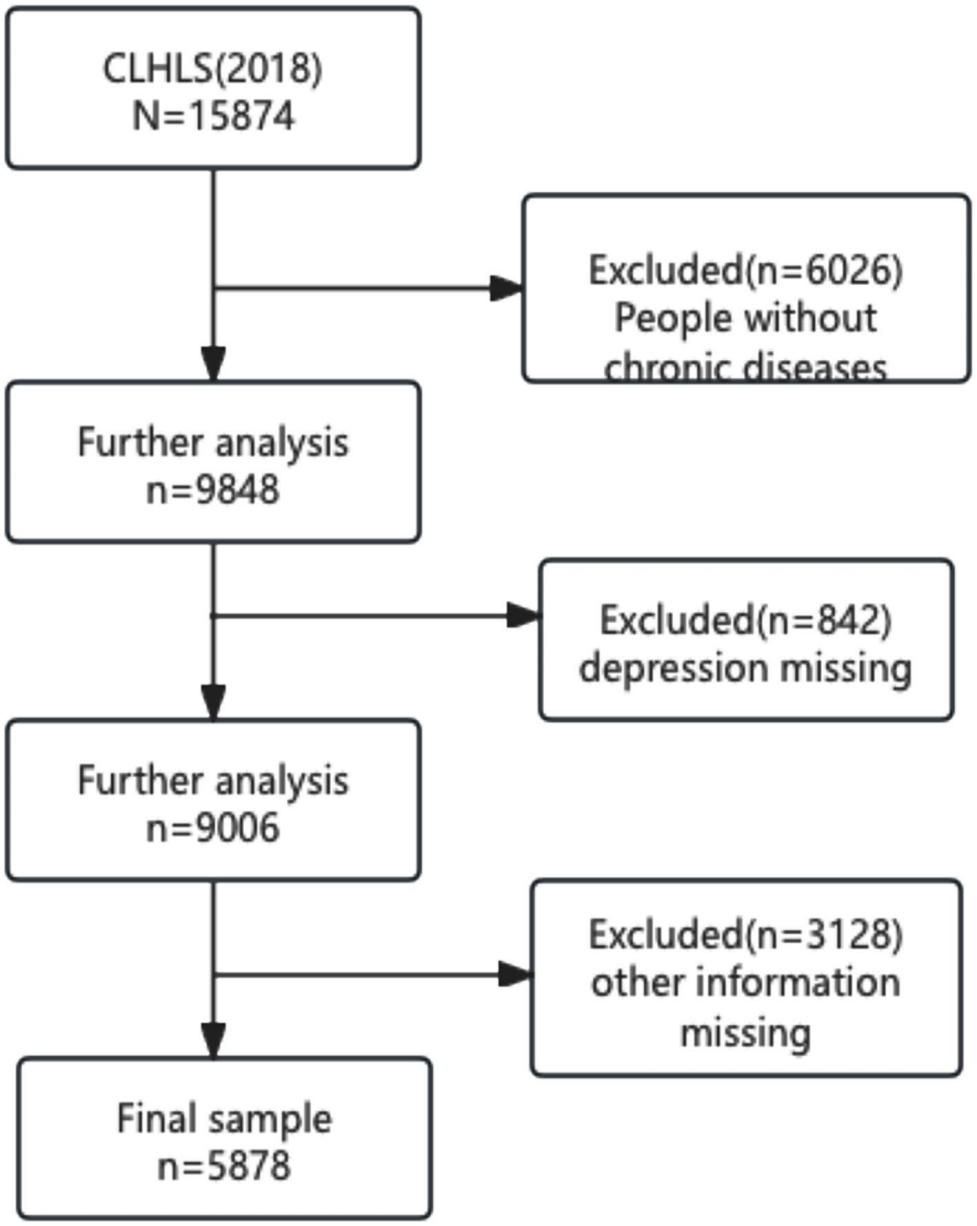
Flow chart of sample selection.

In this study, we focused on older adults with chronic diseases from the CHARLS 2018 dataset (Center for Healthy Aging and Development Research, Peking University, 2020). During the review process, we encountered significant missing data. After screening and excluding samples with missing information, we retained a total of 5,878 samples as the final study population. The screening process is illustrated in Figure 1.

### 2.2 Measurement

#### 2.2.1 Depression assessment

The CHARLS utilized the 10-item short version of the Center for Epidemiological Studies Depression Scale (CES-D) (Andresen et al., 1994) to assess respondents’ depression status. In this scale, items 5 and 8, which state “I am hopeful for the future” and “I am happy,” are considered positive indicators, while the remaining eight items are negative indicators. Responses to each item are categorized as “rarely or not at all,” “not too much,” “sometimes or half the time,” and “most of the time,” with corresponding scores of 0, 1, 2, and 3 assigned. For positive items, the scoring is reversed. The total score is the sum of all 10 items, with higher scores indicating more severe levels of depression. In this study, the Cronbach’s alpha coefficient for the depression scale was 0.823, demonstrating good reliability and validity.

#### 2.2.2 Demographic information

This study included the following variables based on a review of preliminary literature and basic information from the CHARLS questionnaire: (1) Demographic information: age, gender, current residence status, marital status, whether the individual lives alone, and the number of children; (2) Socio-economic information: years of education, self-assessment of life satisfaction, and self-assessment of economic status; and (3) Healthcare information: self-assessment of health status, smoking habits, alcohol consumption, exercise frequency, enjoyment of retirement policies, activity limitations due to health problems, and regular engagement in physical labor. These variables are summarized in Table 1.

**Table 1:** Case of variable assignment.

Variables	Assignment mode
Gender	1 = male; 2 = female
Age	1 = 60–69; 2 = 70–79; 3 = 80–89; 4 = ≥90
Residence	1 = city; 2 = town; 3 = rural
Marital status	0 = no spouse;1 = have a spouse
Education level	0 = none;1 = 0 to 6 years;2 = ≥7 years
Smoke	1 = yes; 2 = no
Drink	1 = yes; 2 = no
Exercise	1 = yes; 2 = no
Live alone	1 = no;2 = yes
only one child	0 = no;1 = yes
Self evaluation of life	1 = good; 2 = fair; 3 = bad
Self-rated	1 = good; 2 = fair; 3 = bad
Economic situation	1 = good; 2 = fair; 3 = bad
Enjoy retirement system	0 = no;1 = yes
Limited in activities	1 = strongly limited; 2 = limited; 3 = no
Physical labor	1 = yes; 2 = no
IADL	Measured value
Anxiety	Measured value

#### 2.2.3 Anxiety assessment

CHARLS employed the 7-item Generalized Anxiety Disorder Scale (GAD-7) to evaluate anxiety symptoms. Each item is scored as follows: 0 for no occurrence in the past 2 weeks, 1 for occurrence on several days, 2 for occurrence more than half the days, and 3 for occurrence nearly every day. A higher total score indicates a more severe anxiety state. The GAD-7 demonstrated good reliability, with a Cronbach’s alpha of 0.895 (Dhira et al., 2021).

#### 2.2.4 Assessment of activities of daily living

The assessment of instrumental activities of daily living (IADL) in CHARLS consists of eight items, such as the ability to visit a neighbor, shop alone, cook, and do laundry. Each item is scored as follows: 1 for independence, 2 for some difficulty, and 3 for inability. A higher total score indicates greater impairment in performing daily activities (Xiang et al., 2018). The IADL scale exhibited excellent reliability with a Cronbach’s alpha coefficient of 0.936.

### 2.3 Statistical analysis

#### 2.3.1 Control and test for common method bias

To address potential common method bias from using the same measurement environment or item context, this study employed the Harman one-factor method. If the number of factors with eigenvalues greater than 1 is fewer than the critical criterion of 40% variance explained by the first factor, serious common method bias is not present (Podsakoff et al., 2003).

#### 2.3.2 Descriptive analysis

Statistical analyses were performed using SPSS 26. Chi-square tests and ANOVA were utilized for comparisons between categories, while logistic regression was applied for multifactorial analysis, using a significance level of *α* = 0.05.

#### 2.3.3 Missing data handling

Prior to latent profile analysis, this study employed a combination of listwise deletion and missing pattern testing for missing data: First, the missing rates for all core variables (CES-D total score and item scores, number of chronic conditions, self-rated health status, etc.) and covariates were calculated. Results showed that the missing rate for all key variables was <5%; Second, Little’s MCAR test determined the missing data type. The test result (*p* = 0.23 > 0.05) indicated the data exhibited missing completely at random (MCAR) characteristics; Third, given the low missing rate and MCAR compliance, listwise deletion was applied to exclude samples with missing key variables, ultimately yielding 5,878 valid cases; Fourth, to validate the robustness of the missing data handling approach, multiple imputation (set imputation cycles = 20) was additionally applied to missing values, and latent profile analysis was repeated. The results were consistent with those from listwise deletion, confirming that the missing data treatment did not affect the core conclusions.

#### 2.3.4 Latent profile analysis

Latent profile analysis (LPA) was conducted using the CES-D-10 scale as an observational indicator, with Mplus software facilitating the analysis. Starting from an initial single-category model, the number of categories was incrementally increased to identify the optimal model. Model fit was assessed using the Akaike information criterion (AIC), Bayesian information criterion (BIC), and sample-corrected Bayesian information criterion (aBIC), with smaller values indicating better fit. The entropy value, ranging from 0 to 1, measured classification accuracy, with values closer to 1 indicating higher accuracy (≥0.8 suggests over 90% accuracy). Likelihood ratio test metrics, including Roe–Mondale–Reuben corrected likelihood ratios and Bootstrap-based likelihood ratios, were employed to compare model fit, with *p* < 0.05 indicating that a model with k categories outperformed one with k-1 categories (Lin et al., 2025).

Model comparison results indicate that AIC, BIC, and aBIC values progressively decrease from Model 1 to Model 5. Both BLRT and LMRT tests yield *p* < 0.001, suggesting that increasing the number of categories theoretically improves model fit. However, Model 4 exhibits the highest entropy value (0.841) among all category models, achieving optimal classification accuracy. Furthermore, all category proportions are ≥5.2%, indicating sufficient sample size and robust parameter estimation. Although the 5-category model showed marginally better fit metrics than the 4-category model, it contained an extremely small category (2.5%) with insufficient sample size to reliably estimate the distribution characteristics of core variables. This increases the risk of model overfitting, and the newly added category lacks independent clinical significance and interpretability. In conclusion, the 4-category model was ultimately determined as the optimal latent profile model in this study.

#### 2.3.5 Association analysis between covariates and latent categories

A two-step approach is employed to address the association between covariates and latent categories: ① Step 1 constructs a baseline LPA model without covariates to identify latent categories based purely on depression symptom heterogeneity; ② After determining the optimal 4-class baseline model, incorporate demographic, health-related, and socioeconomic covariates as predictors into a multinomial logistic regression model. Using the most common latent category as the reference group, analyze the influence of each covariate on depression subtype assignment, calculating odds ratios (OR) and 95% confidence intervals (CI).

## 3 Results

### 3.1 Demographic information

This study included a total of 5,878 subjects, all older adults with chronic diseases. The analysis revealed a predominance of females among those with high levels of depression, primarily within the age range of 70 to 80 years. Older adults with chronic diseases residing in rural areas exhibited higher levels of depression compared to their counterparts living in urban settings. Interestingly, chronically ill individuals who smoke and consume alcohol demonstrated lower levels of depression than those who abstain from these activities. Furthermore, older adults without partners and living alone showed higher levels of depression. Detailed statistical information regarding the remaining demographic variables is provided in Table 2.

**Table 2:** General characteristics of the chronically ill elderly.

Variables	*N* (%)	Low-level	Low level-high despair	Medium level	High level	*χ*^2^/F	*p*
Gender						77.082	<0.001
Male	2,724 (46.3)	996	159	132	241		
Female	3,154 (53.7)	908	145	1,635	466		
Age						34.362	<0.01
60–69	793 (13.5)	292	38	387	76		
70–79	1,777 (30.2)	638	91	851	197		
80–89	1,650 (28.1)	491	85	854	220		
≥90	1,658 (28.2)	483	90	871	214		
Residence						29.583	<0.01
City	1,880 (32)	680	92	911	197		
Town	1,758 (29.9)	538	99	869	252		
Rural	2,240 (38.1)	686	113	1,183	258		
Marital status						84.736	<0.001
No spouse	2,937 (49.9)	821	143	1,531	422		
Have a spouse	2,941 (50.1)	1,083	161	1,432	265		
Education level							
None	2,301 (39.1)	609	102	1,231	359		
0–6	2,059 (35)	704	114	1,023	218		
≥7	1,518 (25.8)	591	88	709	130		
Smoke						12.409	0.006
Yes	863 (14.7)	313	51	418	81		
No	5,015 (85.3)	1,591	253	2,545	626		
Drink						50.659	<0.01
Yes	827 (14.1)	345	52	367	63		
No	5,051 (85.9)	1,559	252	2,596	644		
Exercise						202.869	<0.01
Yes	2,289 (38.9)	959	111	1,064	155		
No	3,589 (61.1)	945	193	1,899	552		
Live alone							
No	4,695 (79.9)	1,614	238	2,348	495		
Yes	1,183 (20.1)	290	66	615	212		
Only one child						5.465	0.141
Yes	585 (10.0)	184	40	282	79		
No	5,293 (90.0)	1,720	264	2,681	628		
Self evaluation of life						719.356	<0.01
Good	2,537 (43.2)	1,160	167	1,110	100		
Far	2,409 (41)	621	96	1,392	300		
Bad	932 (15.9)	123	41	461	307		
Self-rated						820.726	<0.01
Good	2,537 (43.2)	1,160	167	1,110	100		
Fair	2,409 (41)	621	96	1,392	300		
Bad	932 (15.9)	123	41	461	307		
Economic situation						408.49	<0.01
Good	1,298 (22.1)	576	74	580	68		
Fair	4,039 (68.7)	1,239	202	2,147	451		
Bad	541 (9.2)	89	28	236	188		
Enjoy retirement system						39.443	<0.01
No	3,726 (63.4)	1,109	187	1,937	493		
Yes	2,152 (36.6)	795	117	1,026	214		
Limited in activities						260.945	<0.01
No	492 (8.4)	99	23	244	126		
Limited	1,461 (24.9)	333	63	814	251		
Strongly limited	3,925 (66.8)	1,472	218	1,905	330		
Physical labor						8.465	0.037
Yes	4,165 (70.9)	1,328	199	2,118	520		
No	1,713 (29.1)	576	105	845	187		
Anxiety	1.459 ± 2.756	0.325 ± 1.168	0.372 ± 1.255	1.376 ± 2.277	5.265 ± 4.341	796.37	<0.001
IADL	19.500 ± 5.525	20.627 ± 5.022	19.701 ± 5.829	19.284 ± 5.522	17.286 ± 5.918	68.205	<0.001

### 3.2 Results of latent profile analysis

This study performed a latent profile analysis (LPA) based on CESD-10 scale scores, exploring potential subgroups by evaluating models with 1 to 5 categories. The model fit information (detailed in Table 3) revealed progressively lower AIC, BIC, and aBIC values from categories 1 to 5. All models achieved entropy values above 0.8, indicating clear delineation of categories, and both the LMR and BLR tests showed significant results, suggesting an improved model fit with additional categories. However, the five-category model included a category comprising less than 5% of the sample, while the three-category model had an entropy value below 0.8 despite significant LMR and BLR tests, potentially impacting model accuracy. Notably, the four-category model exhibited the highest entropy. Consequently, the study adopted the four-category model, as shown in Table 4, which presents the attribution probability matrix. The average probability of subgroup membership ranged from 90.1 to 91.5%, supporting the validity of the four-category profiling results.

**Table 3:** Indicators for each latent profile of depression among chronically ill elderly.

Profile	AIC	BIC	aBIC	Entropy	LMRT (*p*)	BLRT (*p*)	Proportion
1	167519.938	167653.517	167589.963	1	—	—	1
2	157502.341	157709.389	157610.88	0.805	<0.001	<0.001	0.661/0.339
3	154120.61	154401.127	154267.663	0.815	<0.001	<0.001	0.351/0.522/0.157
4	152813.733	153167.718	152999.299	0.841	<0.001	<0.001	0.324/0.052/0.120/0.504
5	151799.938	152227.392	152024.018	0.839	<0.001	<0.001	0.271/0.051/0.171/0.481/0.025

**Table 4:** Attribution probabilities for each latent profile of subjects.

Profile	Profile 1	Profile 2	Profile 3	Profile 4
Profile 1	0.915	0.012	0.000	0.073
Profile 2	0.054	0.901	0.000	0.045
Profile 3	0.000	0.000	0.911	0.089
Profile 4	0.059	0.011	0.024	0.906

### 3.3 Naming of potential profiles

**Figure 2:**
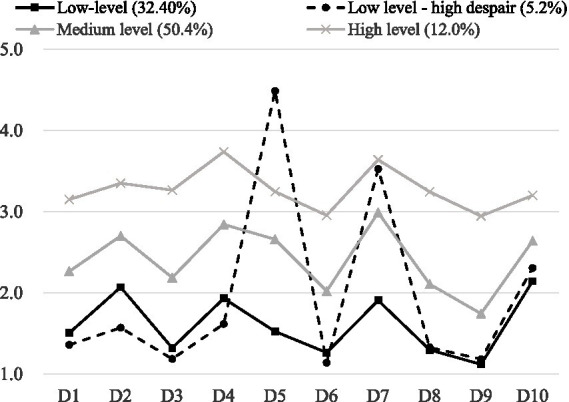
Four types of LPA model profiles.

Based on the latent profile analysis, the average scores and relative levels of the four categories in the CES-D scale were plotted (see Figure 2 for details). The first category, comprising 1,904 individuals (32.4%), exhibited low average scores across all items and was designated the “Low Level Group.” The second group comprised 304 participants (5.2% of the sample). This cohort scored low on all nine other items of the CES-D scale (covering dimensions such as worry, difficulty concentrating, sadness, and loneliness), but scored significantly higher than the other three groups on the fifth item (item content: “I feel hopeless about the future, “which is reverse-scored, meaning higher scores indicate lower hope for the future and stronger despair) was significantly higher than in the other three groups (*F* = 12.78, *p* < 0.001). Based on this core characteristic—low overall depressive symptom levels but specific elevation in the” despair about the future “dimension—this study named this category the” Low-Level-High-Despair Group.” The third category consisted of 2,963 individuals (50.4%) with medium average scores across all items, earning the designation “Medium Level Group.” Finally, the fourth category, with 707 individuals (12.0%), demonstrated high average scores on all items and was named the “High Level Group.” In Figure 2, D1 to D10 represent 10 items of the CES-D scale, assessing dimensions of emotional well-being including worry (D1), concentration difficulties (D2), sadness/depression (D3), perceived usefulness (D4), hope for the future (D5), nervousness (D6), happiness compared to youth (D7), feelings of loneliness (D8), and sleep quality (D9–D10). Scores range from 1 to 5, with higher scores indicating more severe depressive symptoms in the corresponding dimension. The four profiles are defined by distinct score patterns: low-level (32.40%, consistently low scores across all items), low level-high despair (5.2%, low scores on most items but extreme peak in D5), medium level (50.4%, moderate scores across all items), and high level (12.0%, consistently high scores across all items).

### 3.4 Differences in characteristics between profiles

Chi-square tests and ANOVA were utilized to compare differences in single risk factors among older adults with chronic diseases across various depression profiles. The results indicated statistically significant differences (*p* < 0.05) in several factors, including gender, age, marital status, residential status, educational attainment, smoking, drinking, exercise habits, living alone, self-assessment of life satisfaction, self-rated health, self-rated economic status, retirement system coverage, activity limitations, anxiety levels, self-care capabilities, and physical labor involvement. However, no significant differences were found regarding whether participants had one child (*p* > 0.05).

### 3.5 Multiple logistic regression analyses for different depression profiles

Multiple logistic regression analyses were performed with the “Low Level” profile serving as the reference group. The dependent variable was the depression profile classification, while independent variables included those that were statistically significant in univariate analyses. All odds ratios (OR) were adjusted for the covariates listed in the table.

Findings revealed that not living alone [OR = 0.649, 95% CI = (0.462, 0.910)], having average health [OR = 0.559, 95% CI = (0.362, 0.861)], exercising [OR = 0.639, 95% CI = (0.486, 0.840)], engaging in regular physical labor [OR = 0.701, 95% CI = (0.516, 0.953)], and possessing economic affluence [OR = 0.578, 95% CI = (0.340, 0.981)] were negatively associated with being categorized in the “Low Level-High Despair” subgroup.

Conversely, higher levels of anxiety [OR = 1.491, 95% CI = (1.413, 1.572)] and increased activity restrictions due to health problems [OR = 1.286, 95% CI = (1.086, 1.523)] positively predicted categorization in the “Medium Level” subgroup. For the “High Level” subgroup, factors positively predicting membership included higher anxiety levels [OR = 2.052, 95% CI = (1.932, 2.181)], greater activity restrictions [OR = 1.350, 95% CI = (1.023, 1.781)], and not having a partner [OR = 1.743, 95% CI = (1.304, 1.330)].

In contrast, significant negative predictors for the “High Level” subgroup included self-care [OR = 0.965, 95% CI = (0.938, 0.993)], not living alone [OR = 0.578, 95% CI = (0.433, 0.771)], self-rated life satisfaction as good [OR = 0.170, 95% CI = (0.076, 0.397)], self-rated health as good [OR = 0.150, 95% CI = (0.103, 0.219)], and fair health [OR = 0.469, 95% CI = (0.343, 0.641)]. Additionally, exercising [OR = 0.408, 95% CI = (0.343, 0.534)], being economically affluent [OR = 0.345, 95% CI = (0.216, 0.551)], and having an average economic status [OR = 0.554, 95% CI = (0.384, 0.799)] were also significantly negatively predictive of being categorized in the “High Level” subgroup see Table 5 for details.

**Table 5:** Multiple logistic regression analysis.

Variables	Low level-high despair			Medium level			High level		
	OR	LLCI	ULCI	OR	LLCI	ULCI	OR	LLCI	ULCI
Anxiety	0.991	0.873	1.124	1.491^***^	1.413	1.572	2.052^***^	1.932	2.181
IADL	0.986	0.954	1.02	0.989	0.972	1.006	0.965^*^	0.938	0.993
Gender									
Male	0.977	0.726	1.316	0.956	0.822	1.111	0.81	0.619	1.059
Residence									
City	0.73	0.488	1.091	0.882	0.722	1.078	0.984	0.682	1.418
Town	1.139	0.845	1.536	0.941	0.805	1.1	1.215	0.928	1.59
Age									
60–69	0.726	0.433	1.219	0.933	0.719	1.211	0.952	0.585	1.55
70–79	0.812	0.533	1.236	0.907	0.729	1.127	1.007	0.689	1.472
80–89	0.952	0.662	1.37	1.061	0.878	1.282	1.246	0.906	1.714
Live alone									
Yes	0.649^*^	0.462	0.91	0.764^**^	0.637	0.916	0.578^***^	0.433	0.771
Self evaluation of life									
Good	0.354	0.119	1.057	0.552	0.262	1.166	0.170^***^	0.076	0.379
Fair	0.673	0.225	2.012	0.994	0.469	2.108	0.51	0.229	1.134
Self-rated									
Good	0.667	0.43	1.034	0.433^***^	0.341	0.551	0.15^***^	0.103	0.219
Fair	0.559^**^	0.362	0.861	0.758^*^	0.599	0.96	0.469^***^	0.343	0.641
Smoke									
Yes	1.008	0.705	1.443	0.967	0.801	1.168	0.997	0.698	1.426
Drink									
Yes	1.007	0.708	1.433	0.807^*^	0.669	0.972	0.737	0.502	1.083
Exercise									
Yes	0.639^**^	0.486	0.84	0.703^***^	0.613	0.807	0.408^***^	0.313	0.534
Physical labor									
Yes	0.701^*^	0.516	0.953	0.94	0.8	1.104	0.989	0.748	1.308
Limited in activities									
Strongly limited	1.095	0.639	1.877	1.106	0.833	1.467	1.269	0.831	1.936
Limited	1.038	0.74	1.455	1.286^**^	1.086	1.523	1.350^*^	1.023	1.781
Education level									
None	0.807	0.534	1.22	1.114	0.903	1.374	1.132	0.773	1.659
0–6	0.948	0.675	1.332	1.047	0.878	1.249	1.032	0.736	1.448
Enjoy retirement system									
No	0.998	0.694	1.436	1.051	0.872	1.265	0.773	0.55	1.087
Economic situation									
Good	0.578^*^	0.34	0.981	0.832	0.613	1.128	0.345^***^	0.216	0.551
Fair	0.661	0.412	1.06	1.091	0.824	1.444	0.554^**^	0.384	0.799
Marital status									
No	0.877	0.634	1.215	1.168	0.991	1.377	1.743^***^	1.304	2.33

OR, odds ratio; LLCI, lower limit of 95% confidence interval; ULCI, upper limit of 95% confidence interval. Reference group: low-level group. All ORs were adjusted for all covariates listed in the table ^*^p < 0.05, ^**^p < 0.01, and ^***^p < 0.001.

## 4 Discussion

This study categorized subgroups of depressed older adults with chronic illnesses and analyzed factors associated with their depression. The results indicated significant heterogeneity in psychological distress, leading to the rational division of four categories based on model fitting indexes, which confirmed differences in depressive symptoms among older adults with chronic illnesses. This finding aligns with previous studies (He et al., 2023; Martini et al., 2023; Silva et al., 2022; Huang et al., 2022; Chen et al., 2020; Ye et al., 2025).

- Low-level type: In this study, 32.4% of participants fell into this category, characterized by lower depression and anxiety scores. This group appears less prone to psychological distress.
- Low level-high despair type: Comprising 5.2% of participants, this subgroup exhibited negative attitudes toward the future and lower happiness levels. Most individuals were aged 80 or older, suggesting that age may correlate with negative psychological outlooks due to declining health and function (Asante et al., 2022). Economic stability and family support can help maintain some psychological balance, but anxiety and pessimism remain prevalent (Pan et al., 2023).
- Medium level type: Representing 50.4% of the sample, individuals in this group reported higher depression and anxiety scores, coupled with lower self-care abilities. Research indicates that difficulties in self-care contribute to feelings of helplessness, increasing psychological stress (Yin et al., 2023; Kopf and Hewer, 2024). This demographic requires more attention in future research and interventions to prevent progression to more severe depressive states.
- High level type: Comprising 12.0% of participants, this group exhibited the highest levels of depression, predominantly consisting of older adults who were older, lived in rural areas, had lower educational attainment, lived alone, and expressed dissatisfaction with their lives, health, and economic status. Targeted psychological interventions are crucial for alleviating their distress and enhancing quality of life.

The findings also indicated that male chronically ill older adults with spouses, better economic and educational backgrounds, and those who engage in social activities were more likely to belong to the low-risk depression group. In contrast, females were more prevalent in medium and high depression categories, accounting for 66% of those with severe depression. WHO reports that while women live longer, they often face higher rates of illness and disability (Kitas, 2023). Traditional gender roles in China may contribute to women’s higher stress levels, as they typically manage more family responsibilities, leading to reduced personal time and increased psychological pressure (Pinquart and Srensen, 2006; Pinquart and Sörensen, 2011).

As age increases, so does the prevalence of medium and high depression categories. Deteriorating physiological functions and limited daily activities exacerbate fears associated with aging and mortality, leading to negative emotions (Chang and Wang, 2023). Additionally, rural elderly individuals face economic, social, and healthcare disadvantages that contribute to higher depression rates (Wang Y. et al., 2023). Living alone, especially for those without spouses, further intensifies feelings of isolation and helplessness, increasing the risk of depression (World Health Organization, 2023; Yang et al., 2020).

Lifestyle factors also play a significant role; older adults who do not engage in regular exercise are more prone to depression. Exercise not only aids in managing chronic illnesses but also fosters social interaction, reducing feelings of loneliness (Xin et al., 2025; Wang, 2021). Higher scores in instrumental activities of daily living (IADL) (Wang S. et al., 2023) correlate with lower depression likelihood, emphasizing the importance of self-care abilities in maintaining mental health (Sandberg et al., 2019; Casado and Lee, 2012).

Moreover, elderly individuals who perceive their economic status as average or poor (Shi et al., 2023; Sharpe et al., 2023), along with those dissatisfied with their health, are more likely to fall into higher depression categories (Wang, 2020). This highlights the importance of addressing economic concerns and health perceptions in psychological interventions (Ye et al., 2025; Zhong et al., 2021; Xu and Ma, 2020). Lastly, while smoking and drinking were associated with lower depression levels in this study, these behaviors should not be encouraged as healthy coping mechanisms. Instead, promoting positive lifestyle changes, such as regular exercise, is vital for improving mental health among this population.

Although excessive alcohol consumption has been proven to increase the risk of depression in the general population, studies on older adults suggest that moderate drinking may exhibit a “protective effect” through pathways such as social bonding and temporary mood regulation. A similar study using the CHARLS database found that elderly individuals who drank less than once per month had a significantly lower risk of depression compared to those who never drank (95% CI = −0.085 to 0.829, *p* = 0.110) (Xu et al., 2025). This suggests that infrequent drinking may reduce depression levels by alleviating loneliness and enhancing social engagement among the elderly. The European ELSA cohort study similarly noted that moderate drinking following the Mediterranean pattern (<40 g/day for men, <24 g/day for women) did not increase depression risk in older adults and was weakly associated with lower psychological distress scores, consistent with the lower depression levels observed among drinkers in this study (Smith et al., 2024). In this study, smoking was associated with reduced depression severity—not due to any inherent “protective effect” of smoking, but rather the unique characteristics of the elderly population. A study using CHARLS data on middle-aged and elderly Chinese women showed that rural elderly smokers did not exhibit a significantly increased risk of depression (OR = 1.10, 95% CI = 0.78–1.57), while quitters exhibited a trend toward reduced depression risk (OR = 0.77) (Yone et al., 2025). This suggests the observed “smoking-depression association” in this study may represent an apparent correlation driven by confounding factors (e.g., greater social interaction among smokers, reduced perception of pain/discomfort), rather than a causal relationship. Furthermore, studies involving individuals with severe mental illness also confirm that quitting smoking does not increase the risk of depressive symptoms, further supporting that the association between smoking and depression lacks direct causality.

## 5 Limitations

This study has several limitations. First, although the CLHLS sample is nationally representative, regional and selection nonresponse biases may still exist, affecting the generalizability of results. Second, both chronic diseases and depressive symptoms were measured through self-reporting, potentially introducing information bias; moreover, some potential confounders (such as detailed medication history and quality of social support) were not fully collected. Third, despite utilizing longitudinal data, the influence of reverse causality or unmeasured confounders cannot be entirely ruled out. Furthermore, latent class analysis results depend on model specifications, and the limited available indicators in the CLHLS may not fully capture the heterogeneity of comorbidity. Future studies could integrate biomarker data, more frequent follow-up assessments, and causal inference methods to further validate these findings.

## 6 Conclusion

This study categorized the depressive status of older adults with chronic diseases into four distinct subgroups, each exhibiting unique characteristics. Notably, those in the high-level and intermediate-level categories, who demonstrate poorer psychological well-being, require greater attention and effective interventions to alleviate anxiety and depression. Addressing these issues is essential for promoting healthy aging among this vulnerable population.

## Data Availability

The datasets presented in this study can be found in online repositories. The names of the repository/repositories and accession number(s) can be found at: http://opendata.pku.edu.cn/.

## Footnotes

1
http://opendata.pku.edu.cn/
